# PDGFRα/β and VEGFR2 polymorphisms in colorectal cancer: incidence and implications in clinical outcome

**DOI:** 10.1186/1471-2407-12-514

**Published:** 2012-11-12

**Authors:** Purificacion Estevez-Garcia, Angel Castaño, Ana C Martin, Fernando Lopez-Rios, Joaquin Iglesias, Sandra Muñoz-Galván, Iker Lopez-Calderero, Sonia Molina-Pinelo, Maria D Pastor, Amancio Carnero, Luis Paz-Ares, Rocio Garcia-Carbonero

**Affiliations:** 1 Instituto de Biomedicina de Sevilla (IBIS) (HUVR, CSIC, Universidad de Sevilla), Sevilla, Spain; 2 Medical Oncology Department, Hospital Universitario Virgen del Rocio, Sevilla, Spain; 3 Pathology Department, Hospital de Fuenlabrada, Madrid, Spain; 4 Bionostra Aplicaciones Biotecnológicas, S.L.U., Madrid, Spain; 5 Pathology Department, Centro Integral Oncológico Clara Campal, Madrid, Spain

**Keywords:** VEGFR, PDGFR, SNP, Colorectal cancer, Angiogenesis, Prognosis

## Abstract

**Background:**

Angiogenesis plays an essential role in tumor growth and metastasis, and is a major target in cancer therapy. VEGFR and PDGFR are key players involved in this process. The purpose of this study was to assess the incidence of genetic variants in these receptors and its potential clinical implications in colorectal cancer (CRC).

**Methods:**

VEGFR2, PDGFRα and PDGFRβ mutations were evaluated by sequencing their tyrosine kinase domains in 8 CRC cell lines and in 92 samples of patients with CRC. Correlations with clinicopathological features and survival were analyzed.

**Results:**

Four SNPs were identified, three in PDGFRα [exon 12 (A12): c.1701A>G; exon 13 (A13): c.1809G>A; and exon 17 (A17): c.2439+58C>A] and one in PDGFRβ [exon 19 (B19): c.2601A>G]. SNP B19, identified in 58% of tumor samples and in 4 cell lines (LS174T, LS180, SW48, COLO205), was associated with higher PDGFR and pPDGFR protein levels. Consistent with this observation, 5-year survival was greater for patients with PDGFR B19 wild type tumors (AA) than for those harboring the G-allele genotype (GA or GG) (51% vs 17%; p=0.073). Multivariate analysis confirmed SNP B19 (p=0.029) was a significant prognostic factor for survival, independent of age (p=0.060) or TNM stage (p<0.001).

**Conclusions:**

PDGFRβ exon 19 c.2601A>G SNP is commonly encountered in CRC patients and is associated with increased pathway activation and poorer survival. Implications regarding its potential influence in response to PDGFR-targeted agents remain to be elucidated.

## Background

Colorectal cancer (CRC) is the third most common tumour in the world, with over 1.2 million new cases diagnosed every year, and is responsible for about 8% of cancer related deaths [1]. Approximately one third of patients present metastatic disease at diagnosis, and about 40% of those with early-stage tumors will eventually relapse at some point over the course of the disease [2,3]. Although prognosis has greatly improved over the past decades due to significant surgical and medical advances, once the tumor has progressed beyond surgical resectability, the disease is essentially incurable and median survival ranges from 14 to 24 months with best available systemic therapy [4]. Development of new more effective agents is thus actively pursued.

Angiogenesis has become a major target in colorectal cancer therapy. Bevacizumab, a humanized monoclonal antibody against the vascular endothelial growth factor A (VEGF-A), was the first antiangiogenic agent to demonstrate efficacy in CRC. In the pivotal study by Hurwitz et al., the addition of this agent to irinotecan-based combination cytotoxic therapy significantly improved survival compared to irinotecan-based chemotherapy alone in patients with advanced CRC [5]. Subsequently, bevacizumab has been tested in combination with other chemotherapy regimens with more modest results [3,4]. More recently, a benefit in survival has been also reported in patients with advanced CRC with two new promising antiangiogenic drugs: aflibercept (a VEGF trap) in combination with FOLFIRI (folinic acid, 5-fluoruracil and irinotecan) following progression to oxaliplatin-based therapy [6], and regorafenib (a novel tyrosine kinase inhibitor targeting VEGFR, PDGFR, FGFR, RET, KIT and TIE2) as single-agent therapy in patients who had progressed to all standard therapies [7]. These results clearly illustrate angiogenesis inhibition is to play a major role in the management of this disease.

Angiogenesis is a highly controlled process under physiological conditions, such as embryonal development, postnatal growth and wound healing, but is also a critical driver of tumor growth and progression [8]. It is tightly regulated by a complex equilibrium among different pro- and antiangiogenic factors secreted both by tumor cells and by cells of the tumor microenvironment (pericytes, endothelial, mesenchymal or immune cells). VEGF and their receptors represent one of the best validated pathways involved in angiogenesis [9,10]. VEGF stimulates both proliferation and migration of endothelial cells, enhances microvascular permeability, and is essential for revascularization during tumor formation. It is commonly over-expressed in human tumors, and this is often associated with increased vascular density and more aggressive clinical behavior. VEGF-A and its main receptor, VEGFR2/KDR, are key members of this family and common targets of antiangiogenic agents [11,12].

Platelet-derived growth factor (PDGF) and their receptors (PDGFR-α, PDGFR-β and PDGFR-αβ) play also a critical role in angiogenesis regulation by exerting important control functions in mesenchymal cells during development [13]. PDGF is expressed by endothelial cells and acts in a paracrine manner by recruiting PDGFR-expressing cells, such as pericytes and smooth muscle cells, to the developing vessels, thus improving pericyte coverage and vessel function. PDGF signaling promotes cell migration, survival and proliferation and indirectly regulates angiogenesis by inducing VEGF transcription and secretion [10,13,14]. Mutations involving up-regulation of PDGF and/or PDGFR, as well as PDGFR-dependent growth stimulation, have been documented in a number of solid tumors and hematological malignancies, suggesting a likely role of this pathway in carcinogenesis [10,15]. Moreover, agents antagonizing PDGFR-mediated signaling have also demonstrated antineoplastic activity in preclinical models and in clinical trials, including some conducted in patients with CRC (i.e. regorafenib) [7]. Nevertheless, several other drugs also targeting these pathways (i.e. sunitinib, sorafenib) [16,17] have failed to prove a significant positive impact on the outcome of patients with CRC. The biological grounds for these discordant results are not well understood.

Therefore, and in spite of their undeniable success, only a small proportion of patients do actually benefit from antiangiogenic agents, and reliable tools to prospectively identify which patients are more likely to benefit are scarce. In this scenario, efforts to unravel the intricate molecular pathways governing tumor angiogenesis are certainly needed for progress to be made. In the present study, we sought to evaluate the incidence of genetic polymorphisms of some of the key players of angiogenesis, such as VEGFR-2, PDGFR-α and PDGFR-β, and their potential influence in CRC biology. With this purpose we sequenced the tyrosine kinase domains of these receptors in 8 CRC cell lines (T84, LOVO, LS174T, HT29, LS180, SW48, SW480, COLO205) and in 92 tumor samples of patients with colorectal adenocarcinoma. Correlations of encountered genetic variables with protein expression in cell lines, as well as with clinicopathological features and survival of these patients were also analyzed to assess their potential biological and clinical implications.

## Methods

### Laboratory procedures

#### CRC cell lines

Eight human CRC cell lines (T84, LOVO, LS174T, HT 29, LS180, SW48, SW480 and COLO205) were selected and purchased from the European Collection of Cell Cultures (ECACC). They were representative of patients with different gender, age and tumor stage.

#### Cell culture

Each cell line was grown in conditions of temperature, humidity, O_2_ and CO_2_ levels, culture medium and supplements according to providers’ instructions. Once they reached confluence in monolayer DNA extraction was performed. The total DNA yield was determined using a Nanodrop ND-1000 spectrophotometer (Nanodrop Tech, DE, USA).

#### DNA isolation from human tumor samples and culture cells

Formalin-fixed paraffin-embedded tissues from the 92 selected CRC patients were provided by the Pathology Departments of the corresponding institutions. Samples were mainly obtained from the primary tumor (96%), either by surgical (87%) or endoscopic procedures. Three tissue sections of each tumor were first deparaffinized and rehydrated by serial passes in D-Limoneno (Histo-Clear®, National Diagnostic, Atlanta, GA, USA) and ethanol (100%). Then, DNA isolation from both human tumor tissue samples and culture cells was performed with the REAL pure genomic DNA extraction kit (Durviz, Valencia, Spain) according to the manufacturer’s instructions and then purified using ion exchange columns (QIAGEN Miniprep kit Cat. No. 27106). The total DNA yield was determined using a Nanodrop ND-1000 spectrophotometer (Nanodrop Tech, DE, USA).

#### Genotyping

Public databases including National Center for Biotechnology Information (NCBI) ( http://www.ncbi.nlm.nih.gov), University of California Santa Cruz (UCSC) Genome Bioinformatics ( http://genome.ucsc.edu) and Ensembl Genome Browser ( http://www.ensembl.org/index.html) were reviewed to obtain the haplotypes of the three genes of interest and their reported genetic variants. The exomic regions corresponding to the tyrosine kinase domains, which were the regions with the highest probability of mutations, were then identified for each gene: exons 17 to 26 for VEGFR2, and exons 12 to 21 for PDGFRα and PDGFRβ. Specific primers were designed to amplify these exons using expert software in order to minimize non-specific or erroneous amplifications and improve outcomes. Primers used in this study are described in Additional file 1: Table S1. Amplification of the tyrosine kinase domains in both CRC cell lines and tissue samples was performed by a polymerase chain reaction (PCR) method. Fifty nanograms of the genomic purified DNA were amplified in a PCR reaction containing 1.5 units of DNA polymerase EuroTAQ (Genycell Biotech Spain SL; Santa Fe, Granada, Spain), 1xEuroTaq buffer, 2.5 mM Mg^2+^, 0.4 μM forward and reverse primers, 80 μM dNTPs (20 μM each one), 1% DMSO and 1M betaine in a volume of 50 μl. The PCR cycling conditions were as follows: initial denaturation at 94°C for 5 minutes, 5 cycles at 94°C for 1 minute, and annealing that began at 67°C for 45 seconds; this temperature was decreased 2°C each cycle to 59°C (67, 65, 63, 61, 59) and then 45 seconds at 72°C. This was followed by 35 cycles at 95°C 1 minute, 55°C for 45 seconds and 72°C for 45 seconds. The last step was a final extension cycle at 72°C for 10 minutes.

#### DNA sequencing

PCR products were first purified using the microClean kit (Microzone Ltd.; Haywards Heath, UK) or ExoSAP-IT® for PCR Product Clean-Up USB (Affimetrix Inc; Santa Clara, CA, USA) for individual reactions or PERFORMA®DTV V396-Well Short Plates (Genycell Biotech Spain SL; Santa Fe, Granada, Spain) for 96 plate reactions. Direct bidirectional sequencing of the PCR products was done using BigDye®Terminator Cycle v3.1 Sequencing Kit (Applied Biosystems; Carlsbad, CA, USA) and ABI 3110 Genetic Analyser (Applied Biosystems) according to the manufacturer’s instructions. All fragments were double-strand sequenced a number of times, and genetic variations found were checked twice. Sequencing analysis was performed using Chromas Lite, Clustal W and DiAlign software.

#### Analysis of protein expression

Cells were washed twice in 1× PBS, pelleted for 30 seconds at 14000× g and lysed in lysis buffer (Tris–HCl pH 7.5 50 mM, NP40 1%, glycerol 10%, NaCl 150mM, complete protease inhibitor cocktail, 2 mM; Roche). After centrifugation, supernatant protein extracts were aliquoted and stored at −80°C until use. The amount of protein was determined by Bradford assay using BSA (bovine serum albumin) as a standard. The appropriate protein quantity was dissolved in Laemli buffer (Tris–HCl pH 6.8 62.5mM, glycerol 10%, SDS 1%, 2-mercapto ethanol 5%, bromphenol blue 0.0025%) and the proteins were separated in SDS-PAGE gels (12%) before they were blotted onto Nitrocellulose Transfer membrane (Whatman - Protrans). Primary antibodies employed were: p-PDGFR-β (Tyr1021)-R 1:400 (Santa Cruz#sc-12909-R), PDGFR-β 1:500 (Santa Cruz#sc-339), tubulin 1:10000 (Sigma – T6557). The secondary antibodies used were goat anti-rabbit Alexa Fluor 680 1:5000 (Invitrogen – A21057) and donkey anti-mouse IRDye 800CW 1:5000 (Rockland Inc. – 605-731-002).

### CRC study population, tumor samples and data collection

Patients that met the following inclusion criteria were selected for the present study: (1) histologically confirmed diagnosis of primary CRC; (2) adequate clinical data recorded in medical charts; (3) adequate tissue specimen available for additional molecular assays (a proportion of tumor cells > 50% was required). Cases were reviewed according to a previously designed protocol which included the following clinical data: age, sex, date of diagnosis, baseline carcinoembryonic antigen (CEA) plasma levels, primary tumor location, TNM stage [18], histological type, tumor differentiation, surgical treatment (type and outcome of surgery), chemotherapy (adjuvant or for advanced disease, regimen used), radiotherapy (neoadjuvant, adjuvant or palliative), date of last visit or death and cause of death. The study protocol was approved by the institutional review boards of participating centers.

Main characteristics of the 92 included patients are summarized in Table 1 and are representative of a standard CRC population. The median age was 68 years, 63% were male and 40% presented advanced disease at diagnosis. The great majority had conventional adenocarcinomas (86%) and only 13% were poorly differentiated tumors. Cancer specific therapy is outlined in Additional file 1: Table S2. Patients with early stage disease (I-III) underwent primary tumor surgery with curative intent. Adjuvant fluoropyrimidine-based chemotherapy with or without oxaliplatin was indicated in patients with high risk stage II or stage III CRC following surgical resection. Neoadjuvant or adjuvant radiotherapy was added in stage II-III patients with rectum primaries. Patients with advanced stage IV disease were managed primarily with systemic chemotherapy that included oxaliplatin- (44%) or irinotecan-based (13%) combination regimens or fluoropyrimidines alone (3%). With a median follow-up of 31 months (range: 8 to 99 months), 59 patients (64%) had died due to disease progression or to complications of cancer therapy.

**Table 1:** Population and tumor samples characteristics

	**N (%)**
Age (years)	
· Median (range)	68 (45–87)
Gender	
· Female	34 (37.0%)
· Male	58 (63.0%)
Primary tumor location	
· Right colon	27 (29.3%)
· Transverse colon	5 (5.4%)
· Left colon	9 (9.8%)
· Sigmoid colon	18 (19.6%)
· Recto sigmoid colon	14 (15.2%)
· Rectum	19 (20.7%)
Histology	
· Conventional adenocarcinoma	79 (85.9%)
· Mucinous or colloid adenocarcinoma	12 (13.0%)
· Signet ring cell adenocarcinoma	1 (1.1%)
Tumor differentiation	
· Well differentiated	22 (23.9%)
· Moderately differentiated	46 (50.0%)
· Poorly differentiated	12 (13.0%)
TNM stage	
· I	8 (8.7%)
· II	22 (23.9%)
· III	24 (26.1%)
· IV	37 (40.2%)
· Unknown	1 (1.1%)
Baseline CEA (ng/mL)	
· High	33 (35.9%)
· Normal	59 (64.1%)
· Median (range)	3 (0–13.318)

TNM: Tumor, Node and Metastases; CEA: carcinoembryonic antigen.

### Statistical analysis

A minimum sample size of 80 patients was planned to be screened in case no mutations were to be encountered, as in such a case the probability of finding mutations in the general population was estimated to be very low (≤ 4.4%; α=0.05, β=0.80) and therefore non-clinically relevant. Considering an expected drop-out rate of about 10% (technical issues or others), 92 patients were finally selected for study entry. Descriptive statistics were used to characterize the most relevant clinical parameters. The association of categorical clinical or pathological features and mutation type was explored by the chi-squared test or Fisher’s exact test when appropriate. Overall survival (OS) was calculated from the time of histological diagnosis to the date of death (deaths due to surgical complications were censored). The Kaplan-Meier product limit method [19] was used to estimate OS, and differences observed among patient subgroups were assessed by the log rank test [20]. Multivariate analysis using the Cox proportional hazards model [21] was performed to assess the association between mutations and clinical outcome while adjusting for other potential confounding factors such as age, tumor stage, primary tumor location, CEA levels and tumor differentiation. P<0.05 was considered significant. All analyses were performed using the Statistical Package for the Social Sciences software (SPSS 16.0 for Windows; SPSS Inc, Chicago, IL).

## Results

### Characterization of VEGFR2, PDGFRα and PDGFRβ genetic variants

Three genetic variations were identified in PDGFRα (exons 12, 13 and 17) and one in PDGFRβ (exon 19) with respect to the registered wild type (WT) reference sequence (NM006206 and NM002609, respectively), whereas no VEGFR2 mutations were detected. Those encountered in exons A12, A13 and B19 were silent mutations showing nucleotide substitution in the third base of the codon without modifying the codified aminoacide, while the one detected in A17 was an intronic insertion. All of them corresponded to single nucleotide polymorphisms (SNP) previously described in public databases with reference SNP IDs rs1873778, rs10028020, rs246395 and rs2412559, respectively (Additional file 1: Table S3).

### SNPs identified in CRC cell lines

Both SNP A12 and SNP A17 were found in homozygosis in all CRC cell lines. PDGFR-A13 SNP was present in heterozygosis in two cell lines (LS174T and LS180), and PDGFR-B19 presented a SNP in heterozygosis in four of them (LS174T, LS180, SW48 and SW480).

### SNPs identified in CRC patient tumor samples

**Figure 1:**
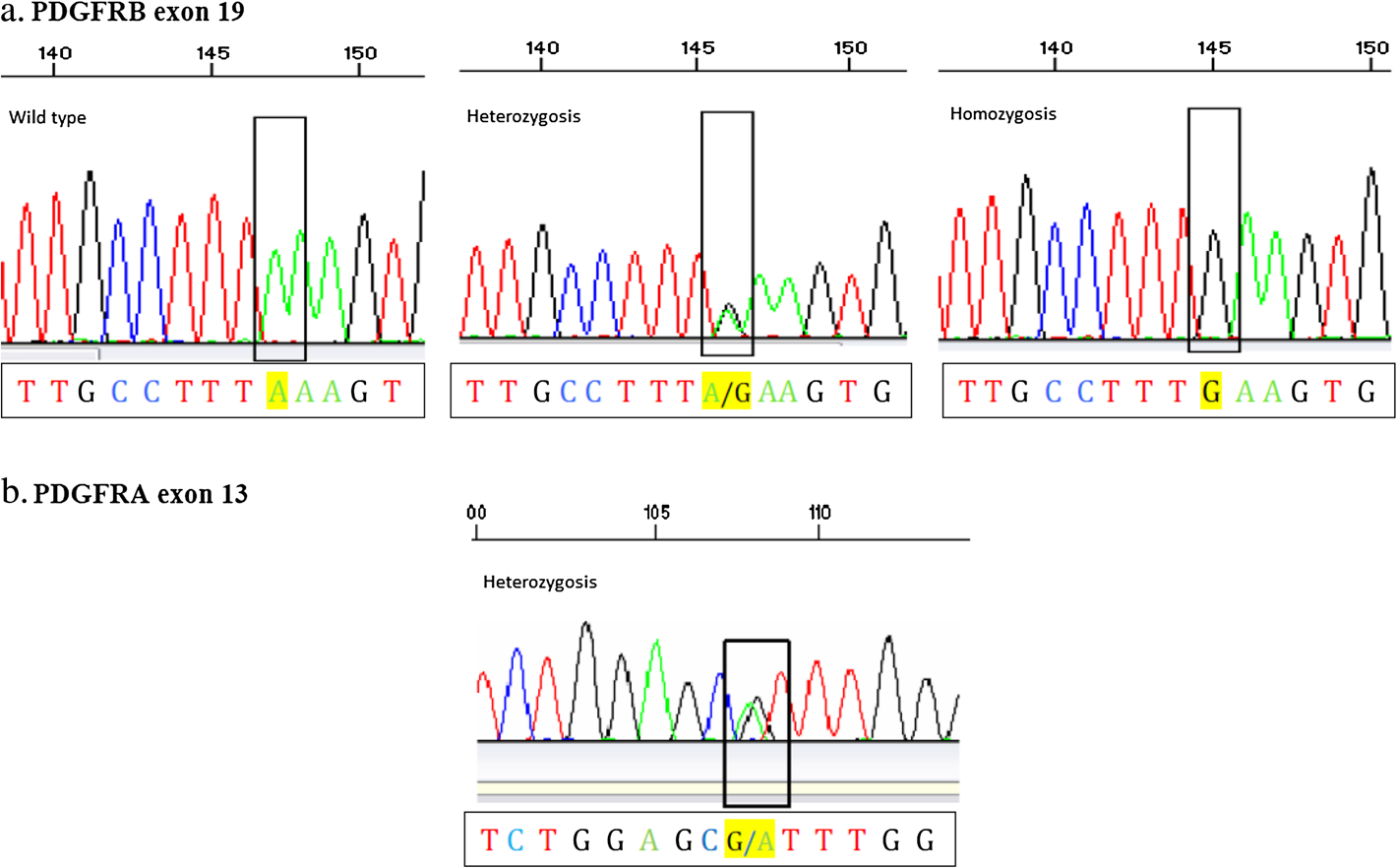
**Electropherogram of a PDGFR sequence. 1a:** Three examples of paraffin-embedded tumour tissue DNA sequence analysis of PDGFRβ exon wild type (Adenine), and SNP B19 in heterozygosis and homozygosis (A to G transition), respectively. **1b:** DNA sequence of a paraffin-embedded tumour tissue sample presenting PDGFRα exon 13 SNP in heterozygosis (G to A transition).

PDGFR-A12 and PDGFR-A17 analysis was feasible in all tumor samples, and all of them showed the SNPs variants in homozygosis. PDGFR-A13 was successfully analyzed in 73 cases (79%), being the SNP A13 detected in heterozygosis in 18% of analyzed samples (13 patients). PDGFR-B19 complete analysis was achieved in 78 patients (85%), and the SNP B19 was found in 58% of evaluable samples (45 patients), both in homo- and heterozygosis (7 and 38 patients, respectively). Figure 1 illustrates DNA sequencing of PDGFRα exon 12 and PDGFRβ exon 19, showing SNPs identified in our population.

### Correlation of PDGFRα and PDGFRβ genetic variants and clinicopathological features

Distribution of SNPs A13 and B19 according to gender, age, baseline CEA levels, primary tumor location, histological type, TNM stage at diagnosis and tumor differentiation is described in Table 2. The only observed correlations that were of borderline statistical significance were those found between SNP B19 and primary tumor location, and SNP A13 and tumor differentiation. Indeed, the PDGFR B19 SNP was more commonly encountered among patients with colon primaries than in those with primary tumors located in the rectum (63.9% vs 35.3%; p=0.051). On the other hand, PDGFR SNP A13 was never detected in well differentiated tumors, whereas it was identified in 23% of moderately or poorly differentiated ones (p=0.053).

**Table 2:** Correlation of PDGFR A13 and B19 mutational status and clinical-pathological features

**Clinical features**	**PDGFRα exon 13**			**PDGFRβ exon 19**		
	**WT N (%)**	**SNP N (%)**	**p**	**WT N (%)**	**SNP N (%)**	**p**
Gender			0.754			0.346
· Female	24 (85.7)	4 (14.3)		10 (34.5)	19 (65.5)	
· Male	36 (80.0)	9 (20.0)		23 (46.9)	26 (53.1)	
Age			0.127			0.653
· ≤ 68-years old (median)	34 (89.5)	4 (10.5)		18 (45.0)	22 (55.0)	
· > 68-years old (median)	26 (74.3)	9 (25.7)		15 (39.5)	23 (60.5)	
CEA (carcinoembryonic antigen)			1.000			0.813
· Within normal range	39 (83.0)	8 (17.0)		21 (41.2)	30 (58.8)	
· ≥ ULN (5 ng/ml)	21 (80.8)	5 (19.2)		12 (44.4)	15 (55.6)	
Primary tumour location			1.000			**0.051**
· Colon	47 (82.5)	10 (17.5)		22 (36.1)	39 (63.9)	
Rectum	13 (81.3)	3 (18.8)		11 (64.7)	6 (35.3)	
Tumour histology			0.401			0.221
· Conventional adenocarcinoma	52 (83.9)	10 (16.1)		30 (45.5)	36 (54.5)	
· Mucinous or colloid adenocarcinoma	8 (72.7)	3 (27.3)		3 (25.0)	9 (75.0)	
TNM stage			0.196			0.170
· I-II	23 (92.0)	2 (8.0)		8 (30.8)	18 (69.2)	
· III-IV	37 (78.7)	10 (21.3)		24 (47.1)	27 (52.9)	
Tumour differentiation			**0.053**			0.586
· Well differentiated	15 (100)	0 (0.0)		7 (36.8)	12 (63.3)	
· Moderately or poorly differentiated	37 (77.1)	11 (22.9)		23 (47.9)	25 (52.1)	
Surgery of primary tumor			0.578			0.389
· Yes	55 (80.9)	13 (19.1)		32 (43.8)	41 (56.2)	
· No	5 (100.0)	0 (0.0)		1 (20.0)	4 (80.0)	
Surgery of metastasis			0.953			0.451
· Yes	13 (86.7)	2 (13.3)		9 (50.0)	9 (50.0)	
· No	47 (81.0)	11 (19.0)		24 (40.0)	24 (40.0)	
Adjuvant chemotherapy			0.295			0.986
· Yes	28 (87.5)	4 (12.5)		14 (42.4)	19 (57.6)	
· No	32 (78.0)	9 (22.0)		19 (42.2)	26 (57.8)	
Chemotherapy for advanced disease			0.683			0.929
· Yes	36 (83.7)	7 (16.3)		18 (41.9)	25 (58.1)	
· No	24 (80.0)	6 (20.0)		15 (42.9)	20 (57.1)	

### PDGFRα and PDGFRβ genetic variants and colon cancer survival

**Figure 2:**
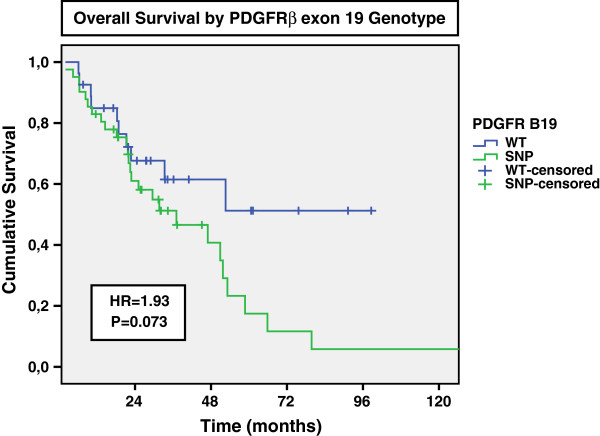
Overall survival of CRC patients by PDGFRβ exon 19 genotype (WT [AA] vs SNP [GA or GG]).

Overall survival of patients according to PDGFR-A13 and -B19 SNPs identified is depicted in Table 3. No significant impact in overall survival was observed for SNP A13. On the contrary, 5-year survival of patients PDGFR-B19 WT was substantially greater than that observed in those harboring the SNP (51% vs 17%; p=0.073) (Figure 2). Multivariate analyses showed the presence of the B19 SNP variant was a significant independent predictor of survival (HR 2.89, p=0.029). Other variable that retained independent prognostic value in the Cox regression model was TNM stage (p<0.001), and age was of borderline significance (p=0.060) (Additional file 1: Table S4).

**Table 3:** Overall survival according to PDGFRα and PDGFRβ mutational status

**Mutational status**	**Patients N (%)**	**Overall survival**			
		**Median (months)**	**% at 5y**	**HR**	**P**
**PDGFR-A13**					
· **WT (AA)**	60 (82%)	37.1	14%	0.96	0.934
· **SNP (AG)**	13 (18%)	21.7	41%		
**PDGFR-B19**					
· **WT (AA)**	33 (42%)	NR	51%	1.93	0.073
· **SNP (AG,GG)**	45 (58%)	37.1	17%		

WT: wild type; SNP: single nucleotide polymorphism; N: number; 5y: 5 years; HR: hazard ratio; NR: not reached.

### Effect of B19 SNP in PDGF receptor levels

**Figure 3:**
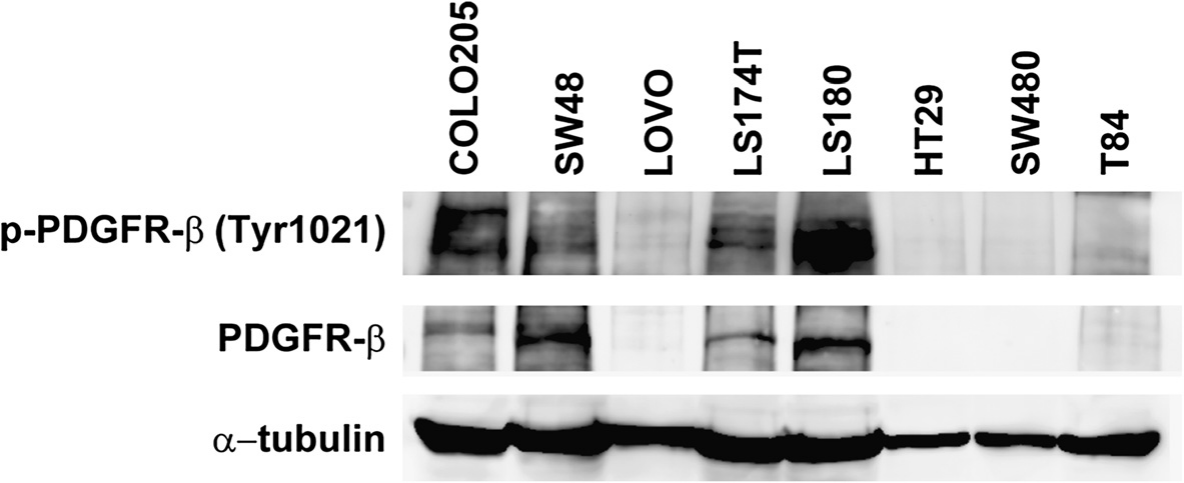
**PDGFRβ protein expression in colon cancer cell lines.** Cell lines that contained the B19 SNP in heterozygosis (LS174T, LS180, SW48 and Colo205) showed higher protein levels of PDGFRβ and p-PDGFRβ.

To explore the potential biological relevance of the identified PDGFR-B19 SNP, we assessed PDGFRβ protein levels in each cell line and correlated them with whether or not they harbored the SNP of interest. Of note, the cell lines that contained the B19 SNP in heterozygosis (LS174T, LS180, SW48 and Colo205) showed higher levels of PDGFRβ protein than those harboring only the wild type allele (Figure 3). In addition, these higher levels of receptor were associated with higher levels of Tyr1021-phosphorylated receptor (Figure 3), indicating its constitutive activation and increased signaling of the pathway.

## Discussion

The present study evaluated the incidence of VEGFR2, PDGFRα and PDGFRβ TK domain genetic variants in different CRC cell lines (T84, LOVO, LS174T, HT29, LS180, SW48, SW480, COLO205) and in tumor samples of 92 patients diagnosed of colorectal adenocarcinoma. Four SNPs were identified, three in PDGFRα [exon 12 (A12): c.1701A>G, rs1873778; exon 13 (A13): c.1809G>A, rs10028020; and exon 17 (A17): c.2439+58C>A, rs2412559] and one in PDGFRβ [exon 19 (B19): c.2601A>G, rs246395]. SNP B19, present in 4 CRC cell lines (LS174T, LS180, SW48, COLO205) and in 58% of patients, had a substantial impact on overall survival, with 5-year survival rates of 51% for patients with PDGFR B19 wild type tumors versus 17% for those harboring the SNP variant (c.2601A>G). This is the first study to analyze the PDGFR genotype in a series of human colorectal cancer and its correlation with different clinicopathological features, and to demonstrate a significant association of a PDGFR SNP with patients’ outcome.

Angiogenesis is a complex process controlled by a number of interconnected signaling pathways, among which PDGF and their receptors play a critical role. Moreover, PDGFR has been the target for many newly developed anticancer drugs, some of them with proven efficacy in CRC (i.e. regorafenib) [7] and some that have failed to demonstrate a benefit in patients with this tumor type (i.e. sunitinib, sorafenib) [16,17]. Despite this, however, only few studies have analyzed the clinical implications of PDGF/PDGFR expression in colorectal cancer. In this regard, Schimanski and cols reported that specific receptor tyrosine kinases (TK) were overexpressed in K-ras mutated CRC [22]. In particular, VEGFR1, VEGFR2 and PDGFRα expression, documented in 95%, 46% and 62% of tested samples, respectively, were significantly linked to K-ras codon 12 or 13 mutations. Whether this could translate into a higher likelihood of responding to TK inhibitors, however, is a matter of speculation. On the other hand, Wheler et al. reported, in a series of 99 human colorectal carcinomas, that co-expression of PDGFRα/β, observed in 57% of tumor samples, was significantly associated with lymphatic metastasis (P=0.007) and advanced tumor stage (P=0.03) [23]. Similarly, high PDGFRβ tumor stromal expression significantly correlated with more aggressive clinical behavior in patients with breast cancer, including high histopathological grade, estrogen receptor negativity, high HER2 expression and shorter survival [24].

Nevertheless, PDGFR genetic variants had never been previously assessed in CRC patients. In our study, four genetic variants were identified, all of them corresponding to SNPs previously reported in public databases. Three of them were silent mutations (A12, A13 and B19) and the other one was an intronic insertion (A17). PDGFRα exon 12 SNP (rs1873778), present in homozygosis in all CRC cell lines and 100% of analyzed tumor samples, has been also described in other neoplasias although in a smaller proportion of patients, including KIT and FLT3 mutation-negative core binding factor (CBFL) acute myeloid leukemias (14% of 35 patients) [25], cervical adenosquamous carcinomas (30% of 30 patients) [26] and gliomas (7% of 86 patients) [27]. In this last study, no association was found between the presence of this mutation and PDGFRα tissue expression. Our results are in agreement with the distribution reported for a European Caucasian population at the NCBI website ( http://www.ncbi.nlm.nih.gov/sites/entrez/), being the G-allele the most frequently encountered (p=0.98). PDGFRα exon 13 SNP (rs10028020), detected in heterozygosis in 2 (LS174T and LS180) of the 8 cell lines examined and in 18% of tumor samples, was associated with poorer tumor differentiation but no significant correlation was found with survival. This polymorphism had been first reported also in heterozygosis by Trojani et al. in 34% of CBFL acute leukemias [25], although potential association of this genotype with clinical features or patient′s outcome was not explored by these authors. Finally, neither PDGFRα exon 17 SNP (rs2412559), identified in all of our patients, nor PDGFRβ exon 19 SNP (rs246395), present in 58% of them, had been previously described in human cancers. PDGFR B19 SNP has been reported to be present in the general population with a frequency of 37%, and was more commonly encountered in our study population among colon primary tumors (64%) than in tumors of rectal origin (35%). Of note, and despite not being an activating mutation, the B19 SNP was found to be a significant prognostic factor (HR: 2.89, p=0.029) independent of tumor stage or patient′s age. This negative effect on patient′s survival did not differ according to primary tumor location (data not shown).

That the identified SNP in exon 19 of PDGFRβ may indeed have relevant biological implications is further supported by the fact that analysis of protein content in cell lines demonstrated the presence of the B19 SNP clearly correlated with higher protein levels of the PDGF receptor β, also in its phosphorylated state. PDGF pathway constitutive activation maintains highly active MEK, thus phosphorylating Bad and inhibiting apoptosis [14,15]. Increased PDGF pathway activation has been also shown to contribute to drug resistance by activating the PI3K pathway [14,15]. Whether or not the presence of this SNP may portend particular sensitivity to PDGFR-targeted agents is a matter of speculation but certainly deserves further investigation due to its relevant potential clinical applications.

On the contrary, no relevant findings were identified in our series regarding VEGFR2 TK domain SNP analysis. As in other solid tumors, overexpression of VEGF mRNA and protein has been associated with tumor progression and poor prognosis of colon carcinoma [28]. The VEGF-A gene is known to be highly polymorphic and harbors numerous SNPs, especially in the promoter, 5’- and 3’-untranslated regions (UTR), which contain key regulatory elements that are sensitive to hypoxia [29]. These SNPs contribute to the high variability in VEGF production among tissues and have been associated with cancer susceptibility, progression, and anti-VEGF therapeutic response in subjects with a variety of solid tumors including colorectal cancer. For example, the 936 T-allele has been associated with increased risk of CRC, advanced stage of disease and worse prognosis, whereas the 634 C allele was predictive of decreased risk and improved survival. SNPs have also been identified in the VEGF receptor genes, although the literature in this topic is still very sparse. Very recently, the VEGFR-1 319 C/A SNP, located in the promoter region of the gene, has been reported to be associated with response to therapy in a cohort of 218 CRC patients treated with different bevacizumab-containing regimens [30]. In this study by Hansen et al., response rates were significantly higher in patients homozygous for the A-allele (AA) than in patients with the C-allele genotype (CC or CA) (56% vs 39%, p=0.015). Similar results were also documented in bevacizumab-treated pancreatic cancer patients [31]. In addition, functional relevance has been demonstrated for several SNPs in the VEGFR-1 and VEGFR-2 genes, particularly SNPs 1192C/T (V2971I; rs2305948) and 1719T/A (H472Q; rs1870377). These SNPs are located in exons 7 and 11, and lead to amino acid changes potentially interfering with the receptor’s binding affinity to VEGF-A. In the current study, however, we aimed to explore potential genetic variations in the TK domain of the VEGFR-2 (exons 17 to 26), which would be expected to have relevant functional consequences. No mutations were however detected in our study population in these gene domains.

Identification of relevant SNPs in critical genes involved in angiogenesis may therefore become valuable tools in assessing risk or predicting cancer response to therapy or prognosis. However, no consensus exists at present regarding the use of any of these for clinical decisions as many studies have reported diverging, conflicting or inconclusive results. Multiple reasons may be responsible for these discrepancies, including gender and interethnic differences in the distribution of alleles, heterogeneous study populations and small sample sizes, different sources of DNA (i.e., tumor vs germline) and different methods for SNP analyses, lack of corrections for multiple testing, links to other loci in the gene or related genes responsible for the observed effect, bias due to post-transcriptional gene regulation, or simultaneous presence of somatic or epigenetic changes that may influence outcome. Prospective validation in appropriately sized and controlled studies is therefore required before these genetic variants may be used in clinical practice.

## Conclusion

In conclusion, the present study has identified, for the first time, PDGFRβ genetic variants with relevant clinical and biological implications. In particular, the G-allele genotype of PDGFRβ exon 19 SNP (rs246395), which was commonly encountered in our series of CRC patients (58%), was associated with increased pathway activation and poorer survival. Further studies to assess the functional consequences of this genetic variant, as well as to validate its role as a prognostic marker in this disease are certainly warranted. Implications regarding its potential influence in response to PDGFR-targeted agents remain to be elucidated.

## Competing interests

The authors declare no conflict of interest.

## Authors’ contributions

Conception and design: RGC, ACM, LPA. Molecular genetic and protein analysis in human samples and cancer cell lines: ACM, JI, SMP, MDP, SM. Pathological assessment: AC, FLR. Collection and assembly of data: ACM, JI, ILC, SMP, MDP, SM, PEG, RGC. Data analysis and interpretation: RGC, LPA, ACM, JI, AC, PEG. Manuscript writing: PEG, SMP, AC, LPA, RGC. Final approval of manuscript: All authors.

## Novelty and impact of present research

This study has identified, for the first time, PDGFRβ genetic variants with relevant clinical and biological implications in colorectal cancer. In particular, the G-allele genotype of PDGFRβ exon 19 SNP (rs246395), encountered in 58% of tumor samples from colorectal cancer patients, was associated with increased pathway activation and significantly poorer survival.

## Pre-publication history

The pre-publication history for this paper can be accessed here:

http://www.biomedcentral.com/1471-2407/12/514/prepub

## Supplementary Material

Additional file 1 Supplementary Tables. Click here for file
