# Supplementary material for: PDGFRα/β and VEGFR2 polymorphisms in colorectal cancer: incidence and implications in clinical outcome
Source: BMC Cancer. 2012 Nov 12;12:514. doi: 10.1186/1471-2407-12-514 (PMC3531259; doi:10.1186/1471-2407-12-514)
Supplement: Additional file 1 — Supplementary Tables. [file 1471-2407-12-514-S1.doc]

**Supplementary Table 2. Oncological therapy**

|  | **N (%)** |
| --- | --- |
| Surgical Treatment of the Primary Tumour  Type of surgery   - Right hemicolectomy - Left hemicolectomy - Subtotal colectomy / Panproctocolectomy - Low anterior resection - Abdominoperineal amputation - Segmental resection / Palliative surgery   Intent of surgery   - Curative - Paliative or diagnostic   Outcome of surgery   - Complete resection - Partial or incomplete resection   Chemotherapy  Type of chemotherapy   - Neoadjuvant - Adjuvant - Metastatic or advanced disease   Adjuvant chemotherapy regimen   - Fluoropyrimidines (FP) - Oxaliplatin and FP (XELOX/FOLFOX)   First-line advanced disease chemotherapy regimen   - Oxaliplatin-based regimen - Irinotecan-based regimen - Fluoropyrimidines alone   Radiotherapy  Neoadjuvant Radiotherapy  Adjuvant Radiotherapy  Palliative Radiotherapy | 83 (90.2%)  23  10  5  25  6  14  60 (65,2%)  23 (25,0%)  55 (59,8%)  28 (30,4%)  2 (2.2%)  36 (39.1%)  55 (59.8%)  33 (35,9%)  3 (3,3%)  40 (43.5%)  12 (13.0%)  3 (3.3%)  3 (3.3%)  10 (10.9%)  8 (8.7%) |

XELOX: Oxaliplatin and Capecitabine; FOLFOX: infusional 5-flurouracyl, folinic acid and oxaliplatin.

**Suplementary Table 3. Characteristics of the PDGFRα and PDGFRβ SNPs identified**

|  | **SNP** | **Allelic change** | **Codified aminoacid** | **Change in aminoacid** | **Rs** | **HGVS Name** |
| --- | --- | --- | --- | --- | --- | --- |
| **PDGFRα** | **12A** | A/G | Proline | No | 1873778 | NM_006206.4:c.1701A>G |
|  | **13A** | G/A | Alanine | No | 10028020 | NM_006206.4:c.1809G>A |
|  | **17A** | C/A | No | -- | 2412559 | NM_006206.4:c.2439+58C>A |
| **PDGFRβ** | **19B** | A/G | Leucine | No | 246395 | NM_002609.3:c.2601A>G |

SNP: single nucleotide polymorphism; Rs: Reference SNP number; HGVS: Human Genome Variation Society

**Supplementary Table 4. Multivariate analysis for Overall Survival**

| **Covariables** | **HR** | **P value** |
| --- | --- | --- |
| PDGFR SNP B19  PDGFR SNP A13  Age  TNM stage  CEA  Tumor differentiation  Primary tumor location | 2.89  0.57  2.75  3.09  0.71  0.85  1.11 | **0.029**  0.348  **0.060**  **0.000**  0.535  0.758  0.378 |
